# Key early proinflammatory signaling molecules encapsulated within circulating exosomes following traumatic injury

**DOI:** 10.1186/s12950-022-00303-0

**Published:** 2022-05-12

**Authors:** Sarah A. Walsh, Thomas A. Davis

**Affiliations:** Department of Surgery, Uniformed Services University of the Health Sciences, 4301 Jones Bridge Road, Bethesda, MD 20814 USA

**Keywords:** Exosomes, Traumatic injury, Inflammation, Blast

## Abstract

**Background:**

Assessment of immune status in critically ill patients is often based on serial tracking of systemic cytokine levels and clinical laboratory values. Exosomes are extracellular vesicles that can be secreted and internalized by cells to transport important cellular cargo in the regulation of numerous physiological and pathological processes. Here, we characterize the early compartmentalization profile of key proinflammatory mediators in serum exosomes in the steady state and following trauma. Adult male Sprague-Dawley rats (91 including naïve) were divided into one of four traumatic injury model groups incorporating whole-body blast, fracture, soft-tissue crush injury, tourniquet-induced ischemia, and limb amputation. Serum was collected at 1, 3, 6, and 24 h, and 3- and 7-day post-injury. Electrochemiluminescence-based immunoassays for 9 key proinflammatory mediators in whole serum, isolated serum exosomes, and exosome depleted serum were analyzed and compared between naïve and injured rats. Serum clinical chemistry analysis was performed to determine pathological changes.

**Results:**

In naïve animals, substantial amounts of IL-1β, IL-10, and TNF-α were encapsulated, IL-6 was completely encapsulated, and CXCL1 freely circulating. One hour after blast injury alone, levels of exosome encapsulated IFN-γ, IL-10, IL-6, IL-13, IL-4, and TNF-α increased, whereas freely circulating and membrane-associated levels remained undetectable or low. Rats with the most severe polytraumatic injuries with end organ complications had the earliest rise and most pronounced concentration of IL-1β, IL-10, TNF-α, and IL-6 across all serum compartments. Moreover, CXCL1 levels increased in relation to injury severity, but remained almost entirely freely circulating at all timepoints.

**Conclusion:**

These findings highlight that conventional ELISA-based assessments, which detect only free circulating and exosome membrane-bound mediators, underestimate the full immunoinflammatory response to trauma. Inclusion of exosome encapsulated mediators may be a better, more accurate and clinically useful early strategy to identify, diagnose, and monitor patients at highest risk for post-traumatic inflammation-associated complications.

## Introduction

Trauma is a leading cause of morbidity and mortality worldwide [1]. Complex polytraumatic injuries occur due to motor vehicle collisions, falls from height, firearm discharges, mass transit collisions and derailments, industrial workplace injuries, terrorist attacks, and natural disasters [2–9]. Injury immediately triggers the body’s complex innate immune inflammatory response, essential for immune surveillance, clearance of debris and necrotic tissue, and preparation for healing and regeneration [10]. However, if unchecked, an overwhelming aberrant immune response at the site of injury can spread systemically to distant organs leading to other life-threatening inflammatory complications, to include systemic inflammatory response syndrome (SIRS), compensatory anti-inflammatory response syndrome (CARS), acute respiratory distress syndrome (ARDS), pneumonia, sepsis and/or multiple organ dysfunction syndrome (MODS) resulting in collateral tissue damage, end organ injury, and increased mortality [10–18].

The innate acute phase immune response to traumatic injury is initially measured by the systemic release of both pro and anti-inflammatory mediators, particularly interleukin-6 (IL-6) [19–21]. However, IL-6 levels peak hours after initial injury, and IL-6 is downstream of other mediators such as tumor necrosis factor-alpha (TNF-α) and IL-1β, limiting early prediction and diagnosis of a patient’s hyperinflammatory state, complications, and outcome [21–23]. Extensive reports have shown that exosomes, small extracellular vesicles (EVs) with a diameter of 30-200 released by all cell types to maintain their regular cellular homeostasis, transfer their molecular cargo to target cells wherein they play a critical role in facilitating intercellular signaling under both homeostatic physiological and pathophysiological conditions [24–34]. Further, exosome-encapsulated cytokines have been reported to be protected from environmental degradation and exert more potent effects than freely-circulating cytokines [35]. Differences in the distribution of free-circulating and EV-encapsulated or EV-associated cytokines have been identified and these differences may be stimulus-dependent [36].

Little is known about exosomes in relation to the steady-state and the early trauma-induced inflammatory immune response. Our objective was to characterize the proinflammatory mediator cargo of serum exosomes in several models of traumatic injury. Specifically, we determined the timing and quantitative level of critical proinflammatory mediators, as well as changes based on insult, and location in the serum compartment, identifying which are freely circulating or exosome membrane-associated, versus those encapsulated in exosomes and undetectable using standard immunoassay methods. We show detection of both free and exosome-associated proinflammatory mediators may be a better, more accurate, and clinically useful early strategy to identify, diagnosis and monitor patients at the highest risk for post-traumatic complications.

## Materials and methods

### Animals

Pathogen-free adult male Sprague-Dawley rats (*Rattus norvegicus*; 400-550 g; 91 animals) were purchased from Taconic Farms (Germantown, New York, USA). All rats were paired and housed in clean standard plastic cages and kept on a 12-h light/dark cycle with unlimited access to food (standard rodent chow), fresh water, and appropriate enrichment. Rats were allowed to acclimate to the vivarium conditions for at least three days before any handling or experimental procedures. After injury, rats were returned to clean home cages with soft bedding. All experimentation involving rats was performed in accordance with institutional standard guidelines and approved (SUR-20-997) by the Uniformed Services University Institutional Animal Care and Use Committee (IACUC) in compliance with all applicable Federal regulations governing the protection of animals in research.

**Fig. 1:**
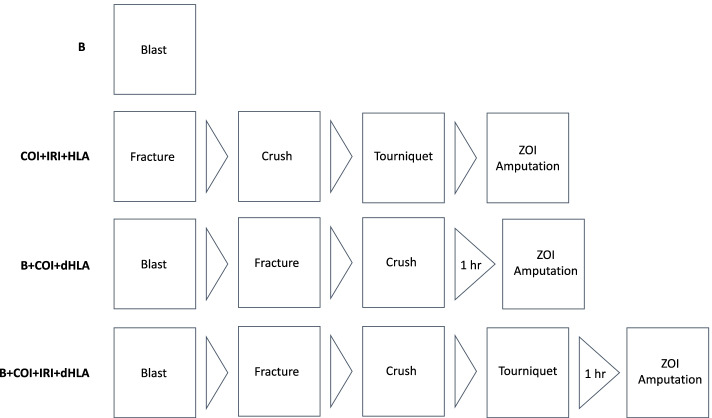
Diagram of experimental groups and sequence of trauma injury patterns**.** Rats were randomly assigned to undergo blast (B), complex orthopaedic injury and ischemia reperfusion injury followed by hind limb amputation through the zone of injury (ZOI) (COI + IRI + HLA), B + COI + 1 h delayed (d) HLA, or B + COI + IRI + dHLA

Rats were divided into one of four experimental groups (*n* = 21/group) (Fig. 1) consisting of (1) blast overpressure exposure only (B); (2) complex orthopaedic injury consisting of right femur fracture and soft tissue crush injury (COI) with 3 h of tourniquet-induced hind limb ischemia and reperfusion injury with tourniquet release (IRI) followed by limb amputation (HLA) through the zone of injury (ZOI); (3) B and COI followed by a 1 h delayed hind limb amputation (dHLA); (4) B and COI with IRI followed by dHLA. Additionally, samples from normal, healthy naïve rats (*n* = 7) were included in the study for comparison.

### Trauma injury patterns

#### Anesthesia

Rats were initially anesthetized with 4% isoflurane and administered a ketamine-xylazine (75 mg/kg ketamine (Henry Schein Animal Health, Dublin, Ohio, USA), 10 mg/kg xylazine (Akorn, Inc., Lake Forest, Illinois, USA)) mixture, with appropriate re-dosing as necessary for the duration of the procedure.

#### Blast overpressure exposure (B)

To induce a whole-body blast injury, rats were secured and placed in an Advanced Blast Simulator overpressure shock tube (ORA Inc., Fredericksburg, Virginia, USA), and exposed to a blast overpressure of 125 ± 10 kPa as previously described [37].

#### Complex orthopaedic injury (COI)

Animals undergoing extremity injuries first had the right hind limb shaved. A Lateral Long Bone Ballistic System (University of Alabama-Birmingham, Birmingham, Alabama, USA) was used to create a comminuted femur fracture in a similar fashion previously described [38]. The right hind limb of the rat was internally rotated and placed between the two anvils on the support stage, then a weight was dropped from a height of 88 cm to reliably produce a mid-shaft comminuted femur fracture. Immediately following the fracture, the anvils and support stage were adjusted to apply a soft tissue crush injury to the fracture site. A pressure of 20 psi was applied for one minute, as determined using a Chatillon DF series force gauge (AMETEK Inc., Berwyn, Pennsylvania, USA).

#### Tourniquet-induced ischemia/reperfusion (IRI)

For pneumatic tourniquet application, a pneumatic cuff (Hokanson, Bellevue, Washington, USA) inflated to 300 mmHg was applied as proximally as possible on the right hind limb to induce prolonged ischemia for 3 h, then released to cause reperfusion injury [39].

#### Hind limb amputation (HLA)

Amputation of the right hind limb was performed through the fracture site with appropriate hemostasis and debridement of devitalized tissue and bone fragments, followed by hamstring and quadriceps myoplasty over the exposed residual femur in a manner similar to that previously described [37, 40].

### Post-operative monitoring

All rats received sustained released buprenorphine (1.2 mg/kg; Zoopharm, Windsor, Colorado, USA) for postoperative pain management, with repeat dosing three days post-injury. Rats were assessed twice daily for three days post-injury using IACUC approved pain charts.

### Serum collection

Whole blood was collected at various timepoints post-injury (*n* = 5-7 rats/injury group). Lateral tail vein sampling occurred at 1 h, 3 h, and 3-day post injury. At 6 h, 24 h, and 7-day post-injury, blood was collected though exsanguination via cardiac puncture. Serum was separated by centrifugation (2000x g, 10 min, room temperature), then aliquoted and stored at − 80 °C.

### Clinical chemistry

Whole serum chemistries were measured using an Element DCSX Veterinary Chemistry Analyzer (Heska, Loveland, Colorado, USA). Kidney function was assessed by measuring blood urea nitrogen (BUN), creatinine (Cr), and the BUN:Cr ratio. Liver function was assessed by measuring alanine aminotransferase (ALT), aspartate aminotransferase (AST) and albumin levels.

### Isolation and characterization of exosomes

Serum exosomes were isolated from 250 μL of freshly isolated whole serum according to manufacturer recommendations using the ExoQuick ULTRA EV Isolation Kit for Serum and Plasma (System Biosciences, LLC, Palo Alto, California, USA). Exosomes were pelleted, and the supernatant was aspirated and saved as the exosome-depleted serum (EDS). The exosome pellet was then re-suspended and subject to column purification for depletion of highly abundant proteins such as albumin and immunoglobulin. EDS and exosome samples were aliquoted and stored at − 80 °C.

Size of isolated exosomes was characterized by dynamic light scattering size analysis using a DynaPro NanoStar and Dynamic 7.9 software (Wyatt Technology Corporation, Santa Barbara, California, USA). The acquisition time of each exosome preparation (4 μL) was five seconds with 10 acquisitions performed in triplicate, and a specific refractive index increment (dn/dc) of 0.185.

Imaging of exosomes was performed by transmission electron microscopy (TEM). Re-suspended exosomes were applied to formvar-coated carbon-stabilized 3 mm copper grids (Electron Microscopy Sciences, Hatfield, Pennsylvania, USA) for one minute and then the excess was wicked away with filter paper. Grids were rinsed gently and briefly in nanopure water to remove buffer salts and 2% aqueous uranyl acetate was then applied for 1 min before the excess was wicked away. Grids were allowed to air dry and then were examined in a JEOL JEM-1011 transmission electron microscope (JEOL USA, Inc., Peabody, Massachusetts, USA). Images were recorded on an AMT XR50 digital camera (Advanced Microscope Techniques, Woburn, Massachusetts, USA).

Protein analysis of whole serum and serum exosomes was performed using SDS-PAGE under reducing and denaturing conditions with protease inhibitors (Promega, Madison, Wisconsin, USA) according to manufacturer instructions (NuPAGE 4-12% Bis-Tris gradient gels; Invitrogen, Carlsbad, California, USA). Sample protein concentrations were determined using a BCA Protein Assay (Pierce BCA Protein Assay kit; Thermo Scientific, Rockford, Illinois, USA).

Exosomal markers were assessed by Western blot analysis of tetraspanins CD9 and CD81, tumor susceptibility gene 101 (TSG101), and programmed cell death 6-interacting protein (ALIX) in total protein preparations of matched serum (10 μg) and serum exosomes (5 μg). Proteins were transferred to a nitrocellulose membrane 30 min using NuPAGE Transfer Buffer (Invitrogen, Carlsbad, California, USA) with a Bio-Rad TransBlot Turbo machine (Bio-Rad, Hercules, California, USA). Nitrocellulose membranes were blocked for one hour at room temperature in blocking buffer (5% BSA (Sigma-Aldrich Co., St. Louis, Missouri, USA) in 0.05% Tween 20 in TBS (Invitrogen, Carlsbad, California, USA)). The membranes were quickly washed with washing buffer (0.05% Tween 20 in TBS), followed by two consecutive washes, then were incubated with primary antibodies in the blocking buffer. Rabbit anti-rat monoclonal antibodies against CD9 (ab109201; Abcam Inc., Cambridge, Massachusetts, USA), CD81 (ab92726; Abcam Inc., Cambridge, Massachusetts, USA), TSG101 (ab133586; Abcam Inc., Cambridge, Massachusetts, USA), and ALIX (ab86429; Abcam Inc., Cambridge, Massachusetts, USA) expression were used at 1:1000 dilution. After overnight incubation at 4 °C and washing, the membranes were incubated with secondary antibody (goat anti-rabbit IgG/HRP conjugate, ab6721; Abcam Inc., Cambridge, Massachusetts, USA) at 1:10,000 dilution at room temperature for one hour, followed by washing. The protein bands were developed using chemiluminescence kit reagents (Immobilon Western Chemiluminescent, Millipore Sigma, St. Louis, Missouri, USA) and visualized on BioRad ChemiDoc (Hercules, California, USA). The intensity of specific bands was quantified by densitometry and Image Lab software (version 6.0.1; BioRad, Hercules, California, USA).

Total protein visualization was performed using a silver nitrate statin of equal total protein preparations of matched serum and serum exosomes. The gel was rinsed in de-ionized water, then placed in a fixative (10% acetic acid, 40% methanol). After fixing, the gel was washed in de-ionized water and sensitized in 12.5% glutaraldehyde. The gel was then washed again in de-ionized water followed by a wash in 20% ethanol. Next, the gel was stained using a silver stain composed of 0.4% silver nitrate, 0.25% ammonium hydroxide, 0.2% sodium hydroxide, and 18.6% ethanol. Following staining, the gel was washed in 20% ethanol, then protein bands were developed using a solution of 20% ethanol, 0.04% formaldehyde, and 0.0006 M citric acid.

### Proinflammatory mediator analysis

At various timepoints post-injury, proinflammatory mediator levels (IFN-γ, IL-1β, IL-10, IL-4, IL-5, IL-6, KC/GRO (CXCL1), IL-13, and TNF-α) in whole serum, isolated serum exosomes, and EDS samples were simultaneously measured using a commercial 9-plex multiplex protein array (V-PLEX Proinflammatory Panel 2 Rat Kits; catalog K15059D; Meso Scale Diagnostics, Rockville, Maryland, USA). Prior to assaying, exosome samples were lysed using a non-ionic lysis buffer (50 mM Tris-HCl pH 7.4, 1% IGEPAL-CA630, 1 mM EDTA, 150 mM NaCl). Samples were analyzed in duplicate, and assays were performed per manufacturer kit instructions. Data acquisition was performed using a Meso Sector S600 (Meso Scale Diagnostics, Rockville, Maryland, USA) and quantitative results were generated using Methodical Mind software (version MMPR 1.0.27; Meso Scale Diagnostics, Rockville, Maryland, USA). Exosome and EDS samples were corrected to account for dilution effects in the isolation process. Quality control and data visualization was accomplished using the Workbench Software (version LSR_4_0_13; Meso Scale Diagnostics, Rockville, Maryland, USA). Data sets from some non-related unpublished studies performed in our laboratory demonstrate that the Meso Scale Diagnostics immunoassay platform used in this study offers a broader linear dynamic range, higher sensitivity, minimal background signals, and significantly less preparatory and processing time with greater throughput and reproducibility than standard ELISA and multi-analyte Luminex (bead-based) immunoassay platforms.

**Fig. 2:**
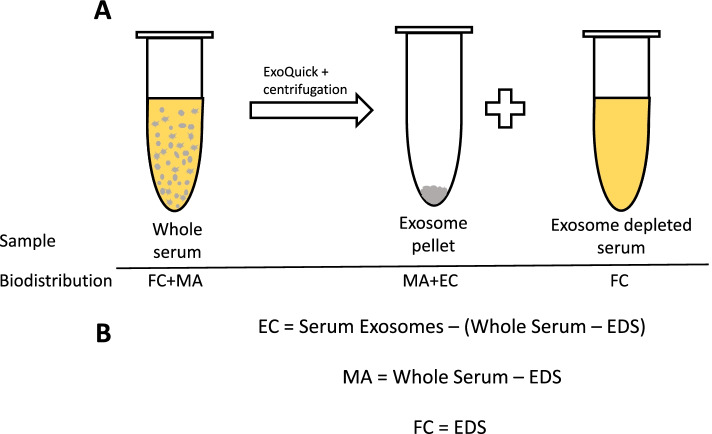
Calculation of concentrations of proinflammatory mediators within each serum compartment. **A** Diagram of serum fraction and associated proinflammatory mediators detected. The gray circles represent exosomes and are not to scale. **B** Equations for calculating proinflammatory mediators in each compartment

In determining the compartmentalization of proinflammatory mediators, the following assumptions were made regarding the detection of mediators in each of the serum fractions:

1. Previous studies have demonstrated that EV-encapsulated mediators are not detected without disruption of the membrane [36]. Therefore, the whole serum measurement illustrated in Fig. 2A represents a standard, conventional immunoassay detection of mediators without treatment with a lysis buffer and the encapsulated mediators are not expected to be detected. The measured whole serum proinflammatory mediators include freely circulating (FC) and exosome membrane-associated (MA) proinflammatory mediators: Whole Serum = FC + MA
2. After precipitation of serum exosomes, measured proinflammatory mediators in the EDS are, by definition, FC. EDS = FC
3. The measured serum exosome proinflammatory mediators include both encapsulated (EC) and membrane-associated (MA) proinflammatory mediators: Serum Exosomes = EC + MA
4. MA and EC fraction cytokine levels must then be calculated from the Whole Serum, Serum Exosome, and EDS measured values (Fig. 2B).

### Statistical analysis

Graphing and statistical analysis were performed using GraphPad Prism (version 9.0.2, GraphPad software, San Diego, California, USA). Outliers were determined using the GraphPad Prism ROUT analysis (Q = 1%). Welch’s t-test was used when comparing two groups. Statistical significance was defined as *p*-value < 0.05.

## Results

### Evidence of systemic injury measured by clinical chemistry

**Fig. 3:**
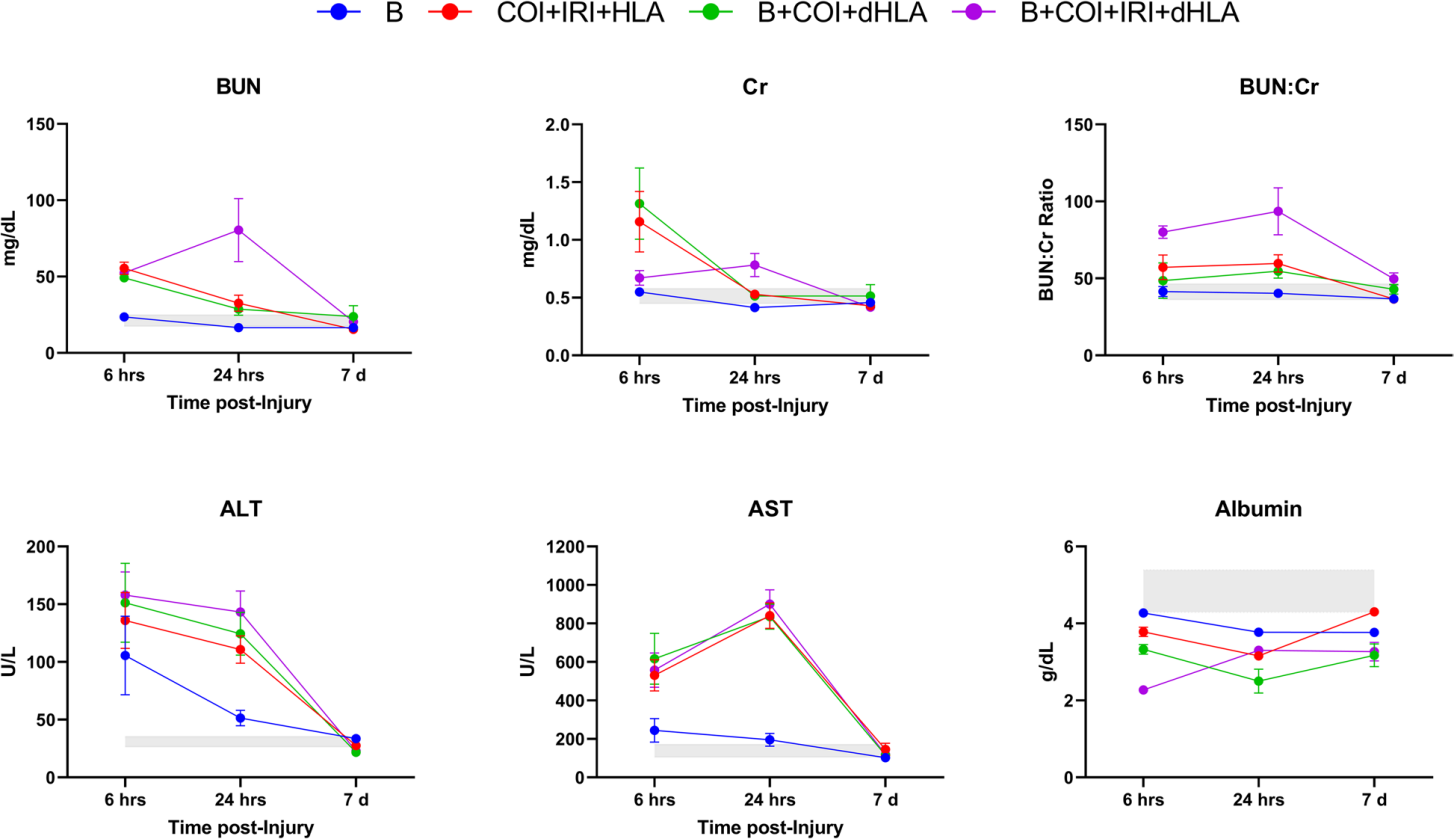
Severity of injury corresponds to acute kidney and liver injury**.** Serum creatinine (Cr), blood urea nitrogen (BUN), BUN/creatinine ratio (BUN:Cr), alanine transaminase (ALT), aspartate aminotransferase (AST), and albumin levels were analyzed using commercial kits at 6 h, 24 h and day-7 post-injury. Data are mean values ± SEM from *n* = 6-7 rats/timepoint. The shaded gray region represents the reference range for normal values (95% CI) from age/weight matched naïve rats (*n* = 7)

We evaluated the levels of clinical chemistry analytes to assess severity of injury in relation to systemic end organ impact, particularly acute kidney injury and acute liver injury (Fig. 3). Blast injury alone had no major effect on serum Cr or BUN levels at any timepoint. As expected, all polytrauma injury patterns caused elevations of BUN and Cr, as well as the BUN:Cr ratio beginning at 6 h post-injury, with significant sustained elevations noted in the B + COI + IRI + dHLA group at 6 and 24 h post-injury (*p* < 0.0001 and *p* = 0.185, respectively). Serum levels of ALT were increased from the normal physiological range at 6 h post-injury in all injury groups, and this increase was significant in groups with IRI (COI + IRI + HLA *p* = 0.0049, B + COI + IRI + dHLA *p* = 0.0007). AST was also increased in all injury groups at 6 h post-injury, but this increase was only significant in the polytrauma groups (COI + IRI + HLA *p* = 0.0027, B + COI + dHLA *p* = 0.0109, B + COI + IRI + dHLA *p* = 0.0032). Hypoalbuminemia was observed in all groups at all timepoints, with the lowest level compared to baseline noted in the B + COI + IRI + dHLA group at 6 h post-injury (*p* < 0.0001). Based on these results, physiological impact of injury patterns, in decreasing severity, is B + COI + IRI + dHLA > B + COI + dHLA > COI + IRI + HLA > B.

### Exosome characterization

**Fig. 4:**
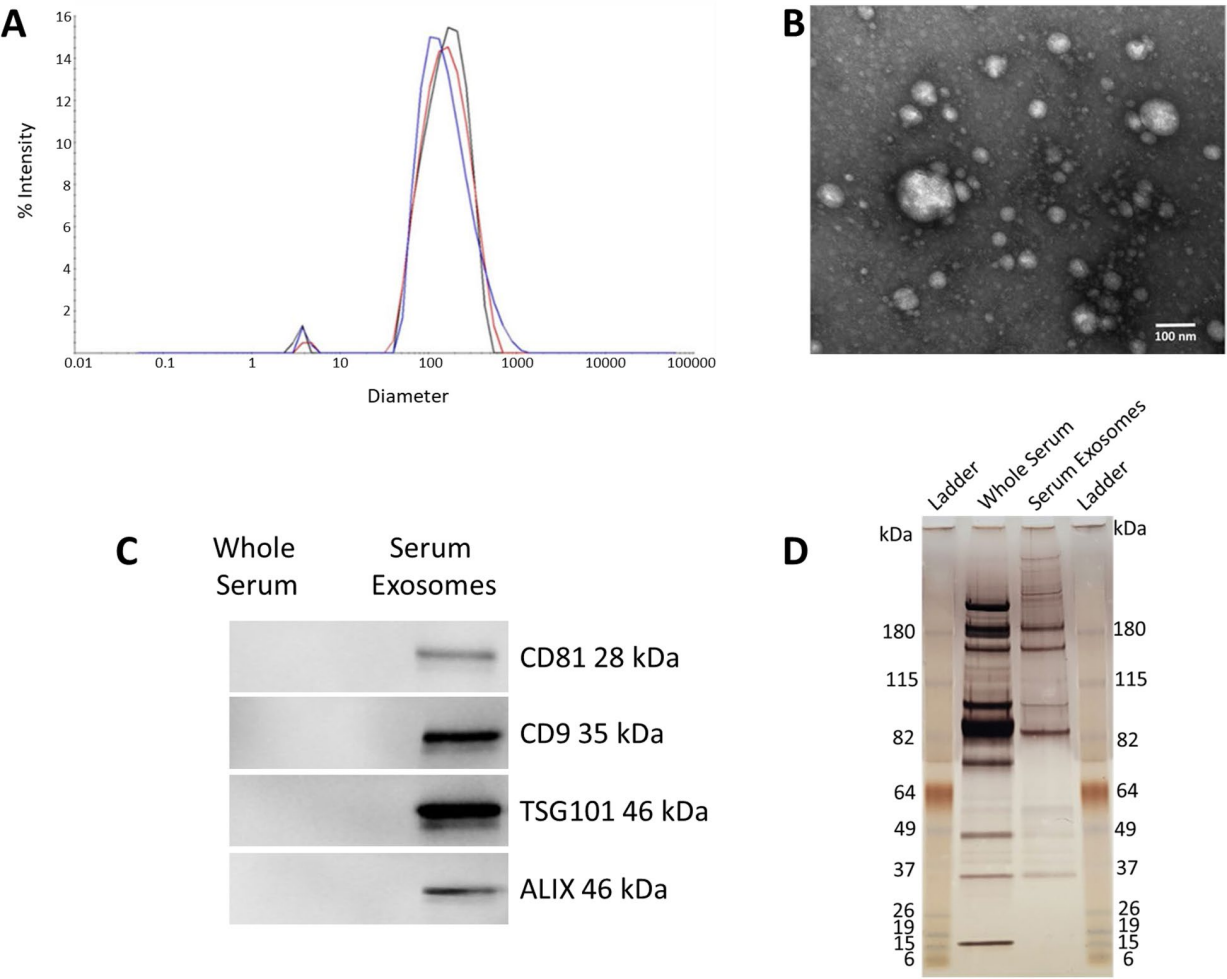
Characterization of serum exosomes. **A** Dynamic light scattering (DLS) results performed in triplicate indicating an average diameter of 164 nm. **B** Transmission electron microscopy (TEM) demonstrating a heterogenous size mixture of discrete vesicles ranging from about 30 – 200 nm. **C** SDS-PAGE analysis of whole serum and serum exosome protein patterns visualized by silver staining. **D** Western blot results showing intense CD9, CD81, TSG101, and ALIX (exosome protein markers) signals in the serum exosomal fraction, but not in the whole serum

The combination of particle size, biochemical, and imaging techniques (DLS, TEM, silver stain, Western blot) indicated successful enrichment and isolation of serum exosomes (Fig. 4). The exosomes had diameters between 30 and 200 nm, with an average size of 164 nm, and contained exosome marker proteins CD9, CD81, TSG101, and ALIX.

### Exosome encapsulated proinflammatory mediators are not detected using conventional immunoassay techniques

**Fig. 5:**
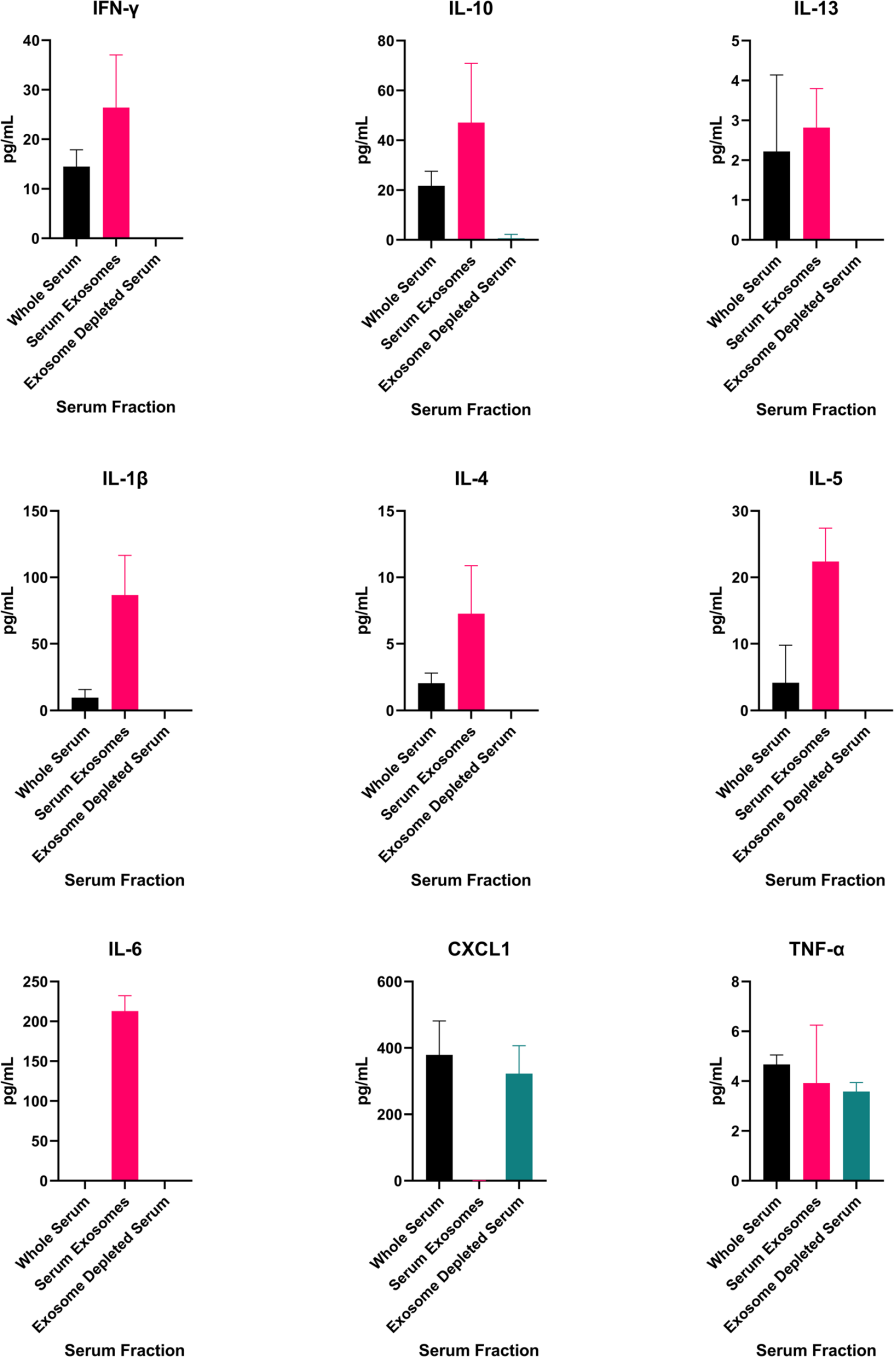
Proinflammatory mediators are predominately exosome-associated in the steady state. Levels of mediators in matched samples from naïve, uninjured rats, of serum, serum exosomes, and exosome depleted serum measured on an electrochemiluminescence multiplex immunoassay. Results represent the mean ± SEM from *n* = 5-7 rats

Using whole serum and isolated serum exosomes collected from healthy, naïve, uninjured rats, we first investigated baseline serum levels and the compartmentalization of IL-1β, IL-4, IL-5, IL-6, IL-10, IL-3, IFN-γ, TNF-α, and CXCL1, which play important roles in driving early innate proinflammatory responses to trauma. We hypothesized that some proinflammatory mediators are encapsulated by the lipid bilayer membrane of exosomes and not detectable by conventional ELISA and multiplex immunoassays. Therefore, in assessing compartmentalization we expected FC and membrane associated MA mediators to be detected in whole serum, EDS to contain only FC analytes, and the serum exosome samples to contain both MA and EC mediators. We developed compartmentalization groupings based on calculated FC, MA, and EC distributions. Mediators detectable after removal of exosomes were classified as “Primarily Freely Circulating.” Mediators not detected in EDS or whole serum were designated “Primarily Exosome Encapsulated” whereas mediators were detected in both whole serum and serum exosomes, but not EDS were classified as “Primarily Exosome Associated” to include both MA and EC. Mediators detected across all compartments were classified as having a “Mixed Distribution.” Similarities in the quantitative levels of mediators between whole serum and EDS for FC mediators indicated that mediators did not co-precipitate in the exosome isolation process (Fig. 5).

CXCL1and TNF-α were all detected in both the whole serum and EDS (Fig. 5). Exosome depletion, removing exosome MA analytes, had no significant effect on CXCL1 assay measurements, indicating that this chemokine is predominantly FC. TNF-α levels in whole serum and EDS were similar, suggesting predominantly FC form. IFN-γ, IL-10, IL-13, IL-1β, IL-4, and IL-5 were detected in whole serum, but not EDS, suggesting that these mediators are MA, but not FC. IL-6 was not detected in either whole serum or EDS (Fig. 5).

**Fig. 6:**
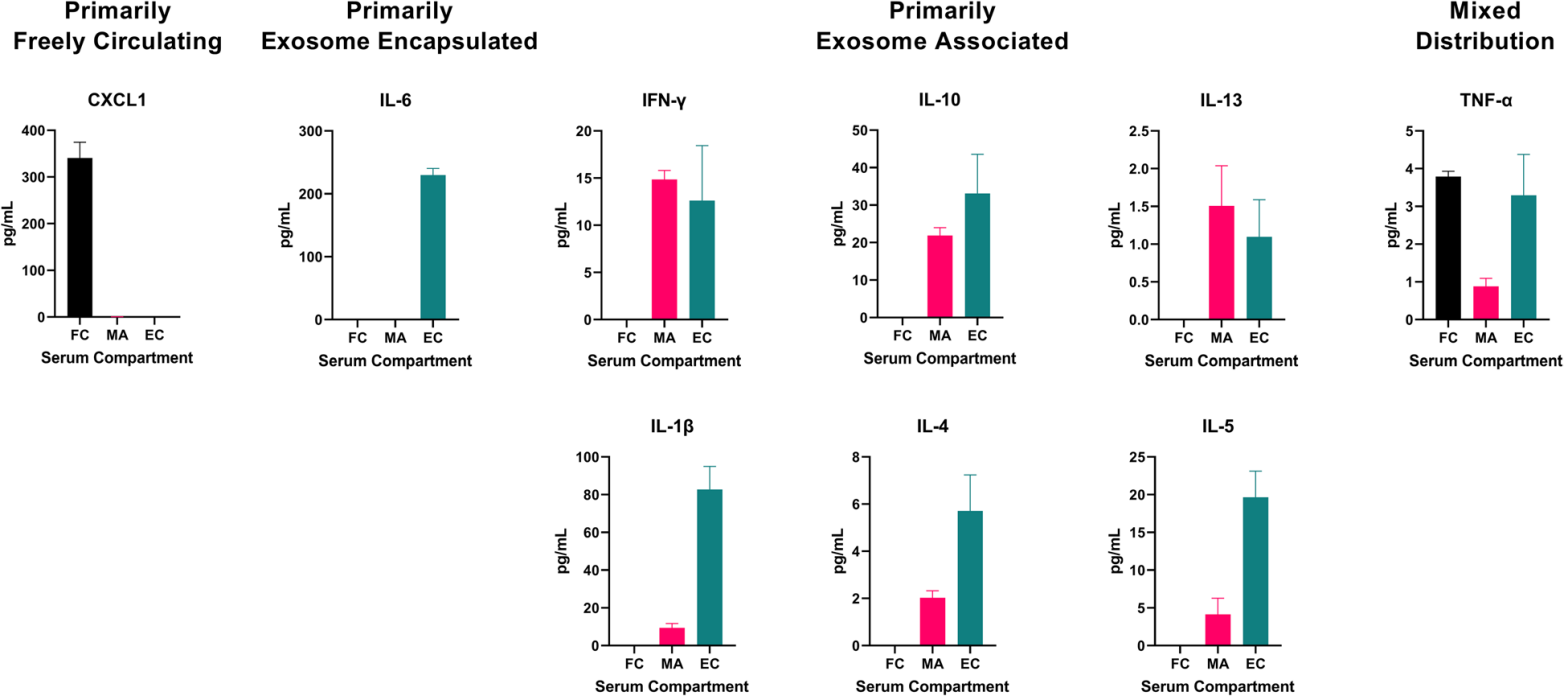
A large proportion of encapsulated proinflammatory mediators in the steady-state evade detection with conventional immunoassays. Serum compartmentalization of proinflammatory mediators in adult male naïve rats according to calculations of freely circulating (FC), exosome membrane-associated (MA), and exosome encapsulated (EC) levels. Results represent the mean ± SEM from *n* = 5-7 rats/timepoint

We next isolated exosomes from fixed serum volumes, then treated the exosome preparations with a non-ionic lysis buffer to disrupt the exosomal membranes and release encapsulated mediators (Fig. 6). Of the 9 proinflammatory mediators tested, an abundance of CXCL1 was detected as only FC. Low to modest levels of MA IL-1β, IL-4, IL-5, IL-10, IL-13 and IFN-γ were detected. We found that there were relatively high levels of proinflammatory mediators encapsulated in exosomes which were not detected in the standard immunoassay procedure in the steady state (Fig. 6). Importantly, an abundance of IL-6 was detected only in the EC compartment in naïve animals. We observed equal or greater amounts of EC IL-1β, IL-4, IL-5, IL-6, IL-10, IL-3, and IFN-γ versus FC or MA, demonstrating these mediators were undetectable using this immunoassay platform without exosomal lysis. After evaluating the exosome preparations, TNF-α was revealed to be equally distributed as a FC and EC cytokine, with a smaller amount in MA form. Our data suggests that most mediators are exosome associated, either encapsulated or membrane-bound, in the steady-state.

### Unique proinflammatory mediator compartmentalization changes following blast overpressure exposure

**Fig. 7:**
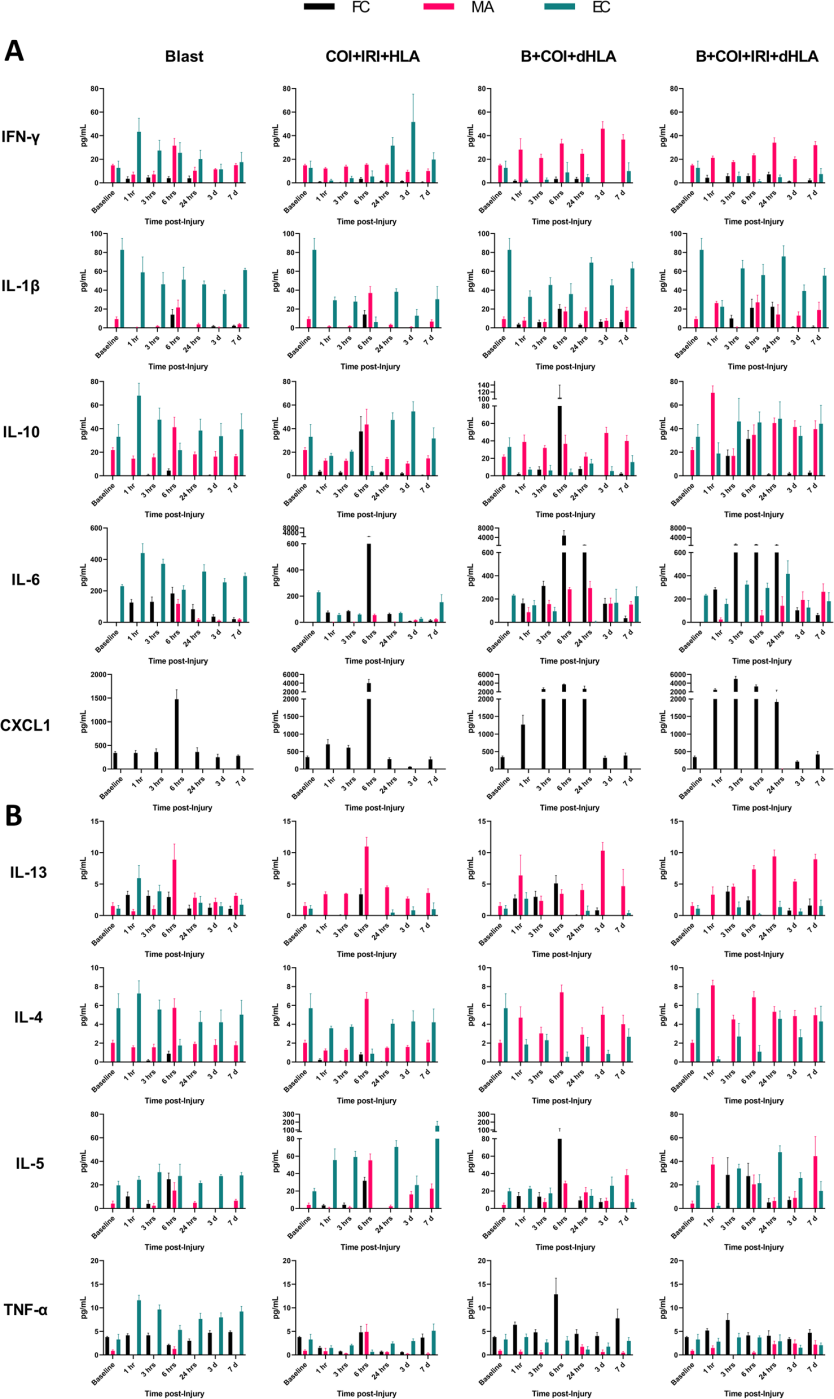
The kinetics of circulating proinflammatory mediator levels and their biodistribution in serum compartments. Compartmentalization values over time post-injury were based on calculation of freely circulating (FC), exosome membrane-associated (MA), and exosome encapsulated (EC) levels following blast (B), complex orthopaedic injury and ischemia reperfusion injury followed by hind limb amputation (COI + IRI + HLA), B + COI + 1 h delayed (d) HLA, and B + COI + IRI + dHLA. Panel-**A** depicts interferon-gamma (IFN-γ), interleukin-1β (IL-1β), IL-10, IL-6, and chemokine (C-X-C motif) ligand 1 (CXCL1) levels in serum compartments. Panel-**B** depicts IL-13, IL-4, IL-5, and tumor necrosis factor-alpha (TNF-α) levels in serum compartments. Data are mean values ± SEM from *n* = 4-7 rats/timepoint. Baseline values are from age/weight matched naïve rats (*n* = 5-7)

After characterizing compartmentalization of proinflammatory mediators in naïve animals, we repeated this procedure in four different models of traumatic injury across six timepoints from 1 h to 7-days post-injury to compare changes in the serum profile as well as the timing and compartmentalization of the same key proinflammatory mediators. After blast exposure alone, serum multiplex analysis demonstrated IFN-γ (1-24 h), IL-1β (6 h), IL-6 (1-24 h), IL-10 (6 h) CXCL1 (6 h), IL-13 (1-24 h), IL-4 (6 h) and IL-5 (1-6 h) levels were low to modestly increased as FC mediators (Fig. 7). At 6 h post-injury, a transient increase in MA IFN-γ, IL-1β, IL-10, IL-6, IL-13, IL-4, and IL-5 was observed. EC IFN-γ, IL-10, IL-6, IL-13, and TNF-α rose sharply at 1 h (*p* = 0.0484, 0.0441, 0.0163, 0.0640, 0.0002, respectively) then decreased gradually returned to near baseline levels at 3 days post injury (Fig. 7). In general, the number of mediators and the percent EC during the first 24 h post-injury was the greatest in the blast alone group.

### Time course changes of proinflammatory mediator compartmentalization and secretion patterns following polytraumatic injury

During the early phase of recovery from polytrauma (1-24 h post-injury) we observed significant changes in the repertoire of FC mediators, specifically higher detectable levels of FC cytokines to include IL-1β, IL-5, IL-6, IL-10, and IL-13 (Fig. 7). The inflammatory chemokine CXCL1, which plays a key role in the innate immune response by recruiting neutrophils, was remarkably measurable only in the FC compartment and was significantly increased within 1 h post injury in all polytrauma groups, with the highest concentrations in the B + COI + IRI + dHLA group, where CXCL1 concentrations peaked by 3 h (4991 ± 1307 pg/ml vs. 340.9 ± 88.7 pg/ml in naïve controls; *p* = 0.0013) (Fig. 7). IFN-γ and IL-4 remained primarily exosome associated across all injury patterns and timepoints (Fig. 7). By day-7, all mediators returned to similar baseline compartmentalization profile patterns, albeit with a tendency towards higher levels than at baseline, with the noted exception of persistent low levels of FC IL-6 across all polytraumatic injury patterns. Interestingly, there was an increase in EC IL-6 similar to that of the blast only data noted in the B + COI + IRI + dHLA, beginning at 3 h (323.4 ± 71.5 pg/mL vs 229.9 ± 23.6 pg/mL in naïve controls; *p* = 0.0402) (Fig. 7).

### Utilization of calculated total cytokine levels increases detection over standard whole serum measurements

**Fig. 8:**
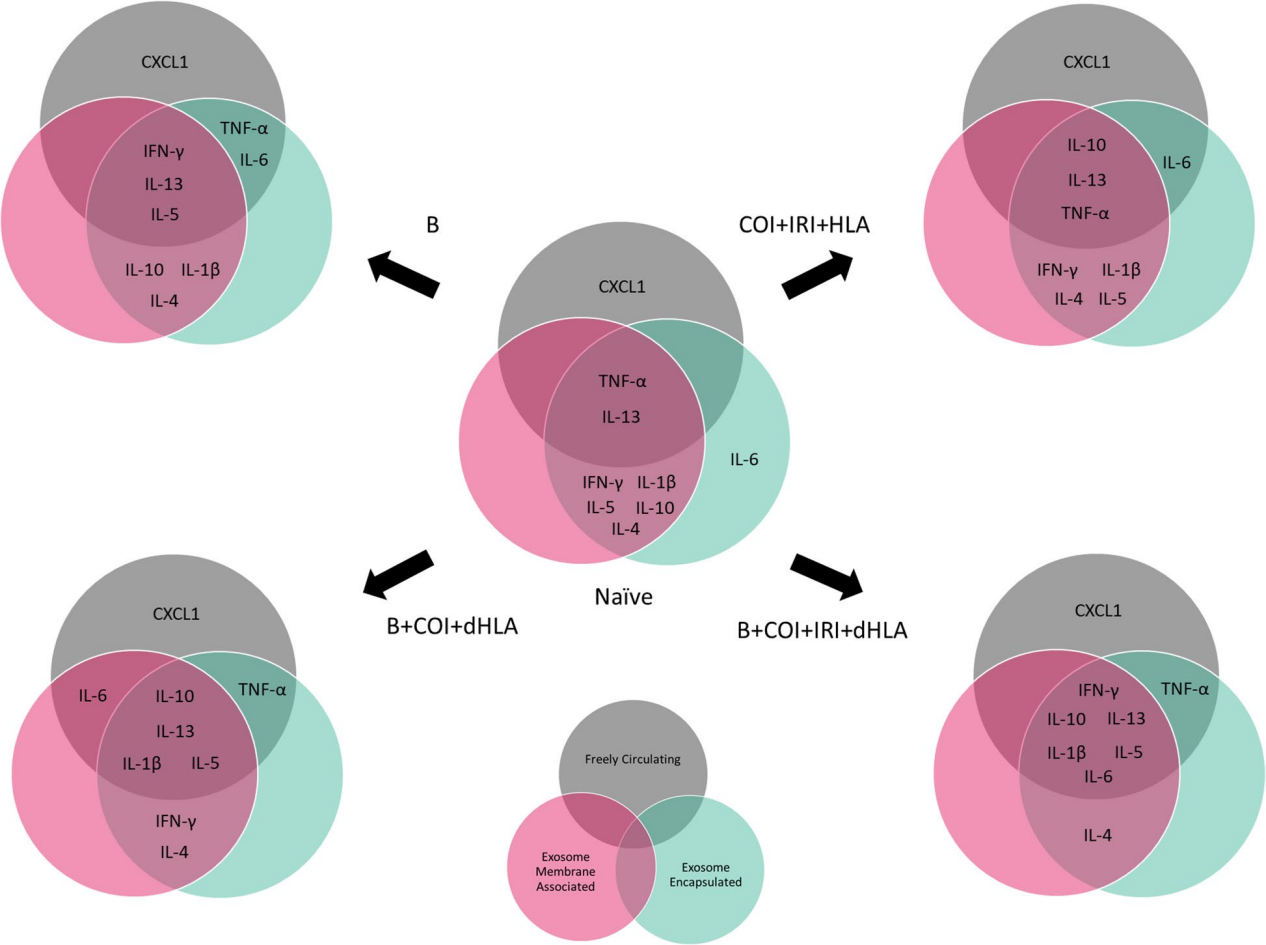
Summary of early proinflammatory mediator compartment changes following traumatic injury. This diagram represents the general early mediator compartment changes in the first 24 h following the four different patterns of traumatic injury based on the results shown in Fig. 7

**Fig. 9:**
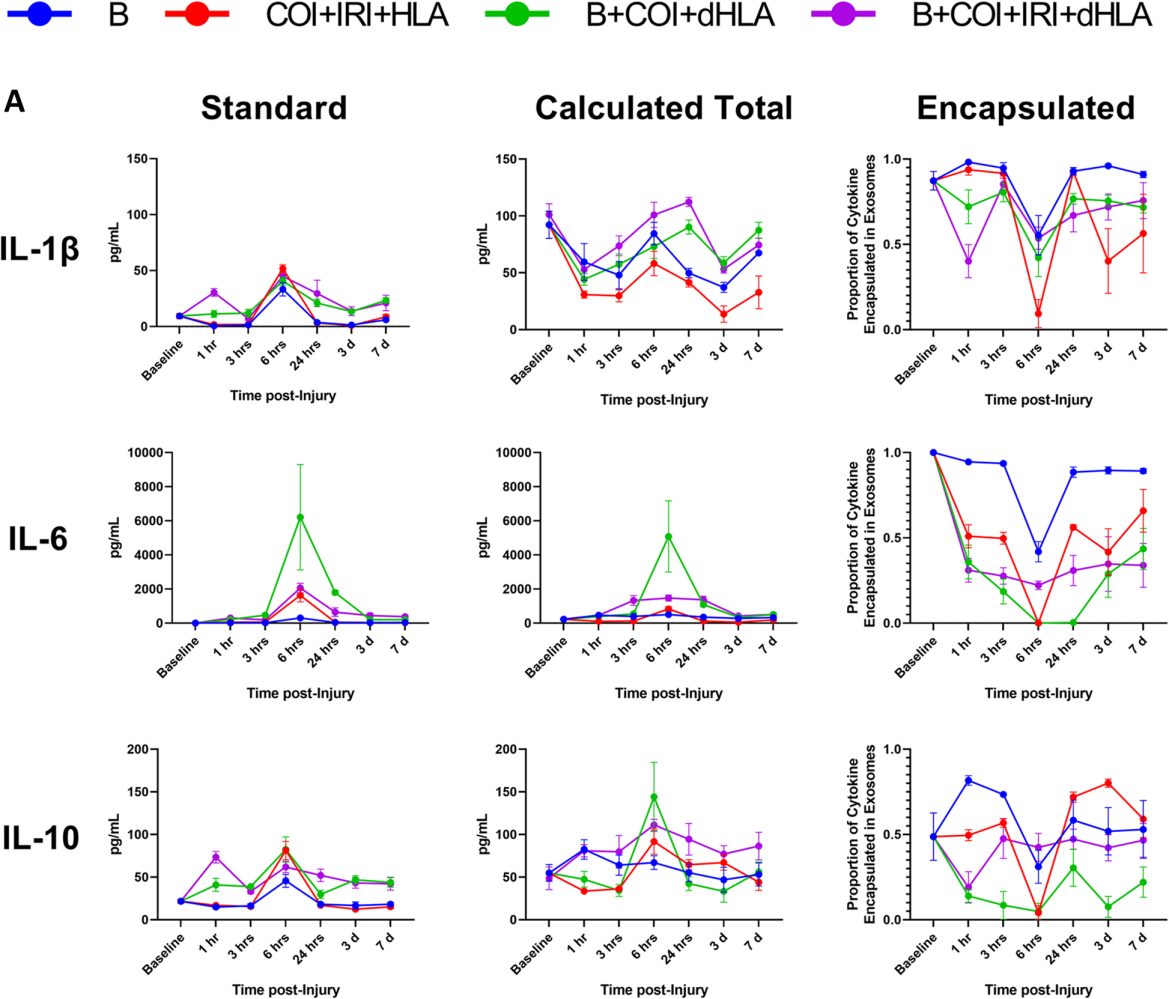

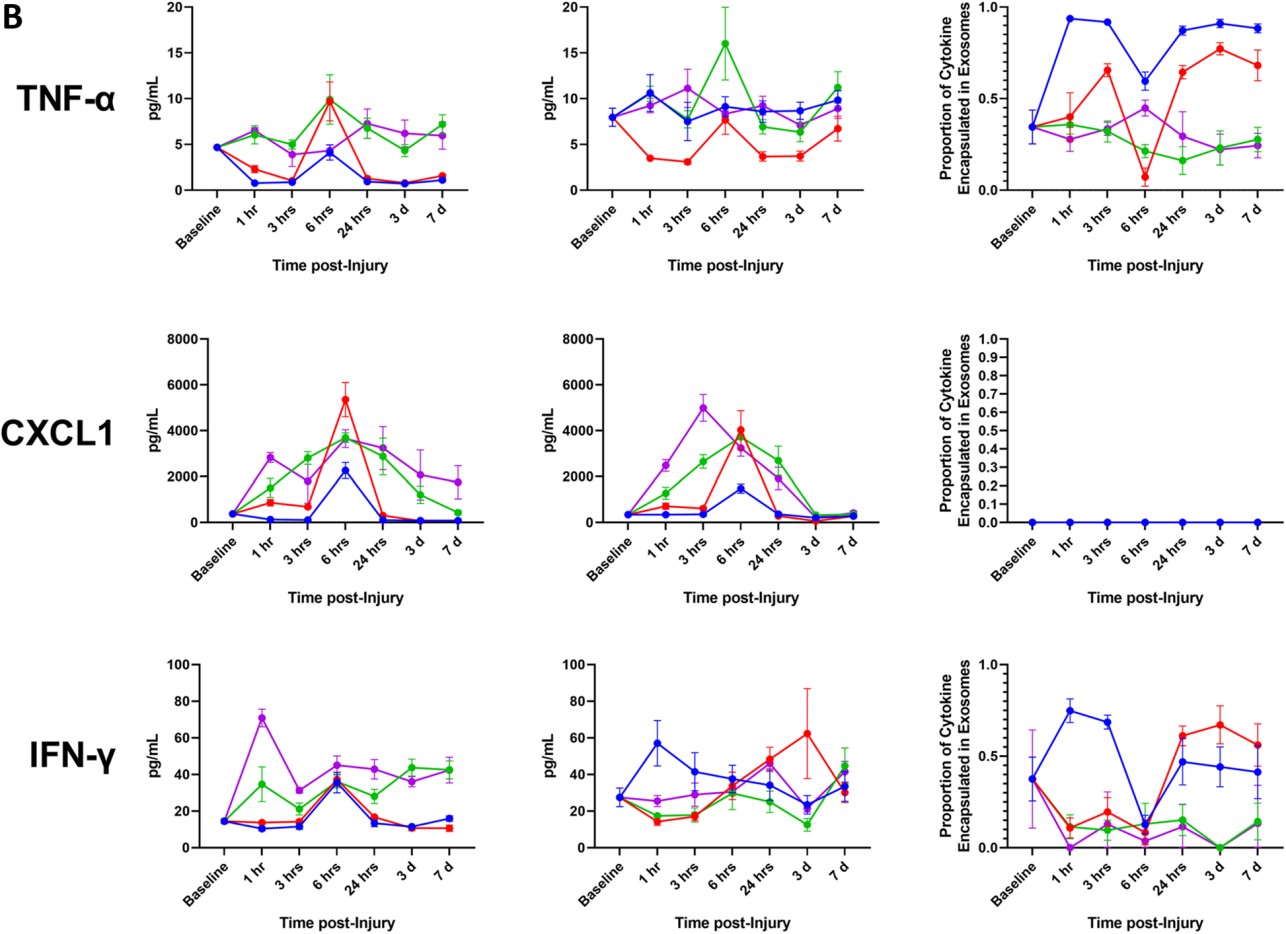

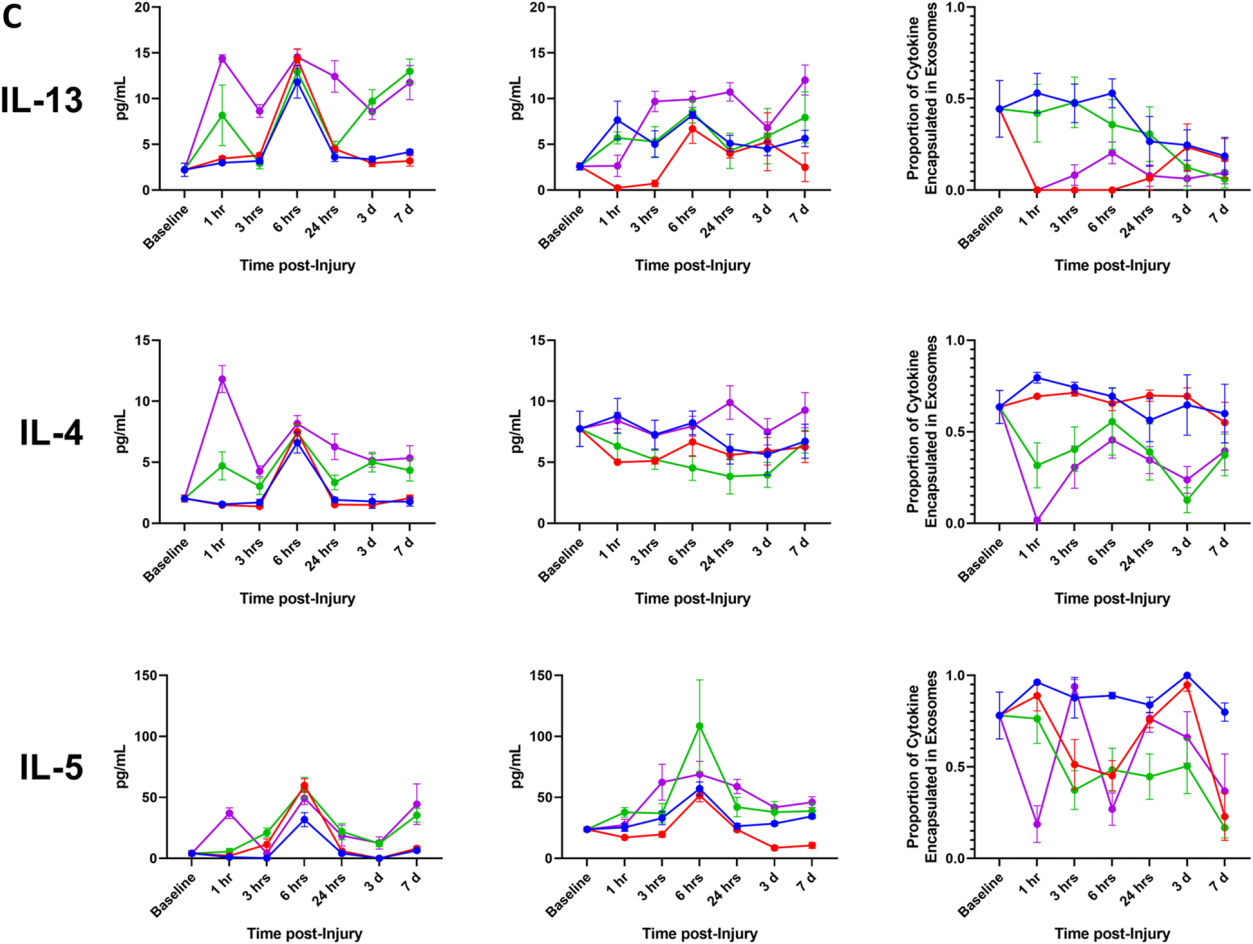
Conventional immunoassay measurements underestimate total proinflammatory mediator levels due to an abundance of encapsulated factors. Comparison of measurements of whole serum and calculated total mediator levels accounting for encapsulated mediators, as well as proportion of encapsulated mediator relative to total mediator level over time after Blast (B), complex orthopaedic injury followed by ischemia reperfusion injury, then hind limb amputation (COI + IRI + HLA), B + COI + 1 h delayed(d)HLA, and B + COI + IRI + dHLA injury patterns. Panel-**A** interleukin-1β (IL-1β), IL-6, and IL-10. Panel-**B** depicts tumor necrosis factor-alpha (TNF-α), chemokine (C-X-C motif) ligand 1 (CXCL1), and interferon-gamma (IFN-γ). Panel-**C** depicts IL-13, IL-4, and IL-5. Results represent the mean ± SEM from n = 4-7 rats/timepoint. Baseline values are from age/weight matched naïve rats (*n* = 5-7)

We have demonstrated that a significant fraction of the assayed proinflammatory mediators are carried as exosomal bound encapsulated cargo both in the steady-state and following traumatic injury. Using Venn diagrams, we illustrate the profile and early compartmentalization changes of these key proinflammatory mediators following traumatic injury (Fig. 8). Therefore, conventional ELISA-based immunoassays no doubt provide an underestimate of the actual biological response. To illustrate this point, we graphed our findings based on measurements obtained using the immunoassay kit versus a calculated total measurement (EC + MA + FC) when encapsulated mediators are considered. In addition, we plotted the proportion of each mediator that is found in the encapsulated form at various time points post injury (Fig. 9).

We observed a re-distribution of IL-6, IFN-γ, IL-4, IL-13 in the first 24 h post-injury, with a less robust response noted for IL-1β and IL-10. The early decrease in proportion of encapsulated mediators corresponded with a delayed increase in detected levels of FC mediators. These changes corresponded in magnitude to the severity of injury. While certain trends were consistent across trauma patterns, such as the sharp decline in proportion of EC IL-1β, IL-6, IL-10, TNF-α, and IFN-γ at 6 h, there were important kinetic differences between trauma patterns. In the blast group, the proportion of EC mediator increased for all mediators except CXCL1, an observation unique to this injury pattern. The combination of blast and COI, without considering IRI status, caused sustained depression of the proportion of EC IL-1β, IL-6, IL-10, TNF-α, and IFN-γ, while blast only or COI + IRI + HLA rebounded to higher than pre-injury levels by day-7. IL-13 had a sharp decrease at 1 h post-injury in trauma patterns including IRI, but a more gradual decline in trauma patterns without IRI.

## Discussion

This study was aimed to better understand the local and systemic immune response to trauma. While commercially available ELISA and multiplex immunoassays are conventionally used to measure the innate immune response to trauma by assessing the concentrations of proinflammatory mediators such as cytokines in peripheral blood, our results indicate that these measured parameters are underestimated, inaccurate, and fail to provide a true picture of the early post-traumatic inflammatory response to trauma. To our knowledge, this is the first qualitative and quantitative profile study demonstrating differential serum compartmentalization of key proinflammatory mediators prior to and following traumatic injury. Depending on the physiological state of the animal (healthy versus type and extent of the injury), significant differences were observed in the compartmentalization (freely circulating versus exosome-associated), pattern of release and concentrations of proinflammatory cytokines like IL-1β, IL-6 and TNF-α. Our findings are consistent with those of Fitzgerald et al. [36] and indicate that a significant number and high levels of “hidden cytokines” are encapsulated within the lipid bilayer membrane of exosomes and thus are not detectable by conventional ELISA and multiplex immunoassays in the absence of vesicle dissociation within samples. We speculate that these exosome-encapsulated mediators play an important yet ill-defined role in early post-traumatic inflammatory response cytokine delivery-signaling and are critical in the regulation of differential physiological and pathological responses. Packaging of cytokines into exosomes may indicate importance of cytokine signaling and delivery, as the lipid bilayer of exosomes gives them the ability to shield their cargo from the microenvironment, leading to stability of the bioactive material within, and increased efficiency of delivery to effector targets [41–46]. Therefore, monitoring total cytokine levels should be done routinely as a new approach to assess patient status and earlier diagnosis of severe post-traumatic complications.

In assessing the serum compartmentalization of proinflammatory mediators in naïve animals in a normal physiological state, we identified a set of encapsulated mediators, undetected using a conventional multiplex immunoassay, at moderate to high levels. The encapsulated mediators are likely sequestered and presumably not immediately active while circulating in the serum, whereas freely circulating and membrane associated mediators have direct access to receptors expressed on cell membranes. The identification of high levels of sequestered IL-6 suggests exosomes may serve a role as a reservoir for immediate release in activation of the innate immune response, and this is supported by the re-distribution phenomenon noted in the polytraumatic injury patterns. IL-6 is not the first mediator released in response to trauma – induction of IL-6 is dependent on TNF-α and IL-1β, therefore, a sequestered ready reserve would be valuable in the response to injury, especially to activate hepatocytes for early release of acute phase proteins [19]. Similarly, we found IL-1β is mostly encapsulated, but a small proportion is immediately accessible, albeit membrane bound. Since TNF-α is a more immediate responder with a short half-life, it is conceivable that it would be found across all compartments at low levels. CXCL1 is a critical chemoattractant involved in recruitment and activation of neutrophils and macrophages during the innate immune response, but also has been reported to function in NLRP3 inflammasome activation, reactive oxygen species formation, and neutrophil extracellular trap formation, suggesting a need for easily accessible basal levels for immediate response [47].

Systemic blast overpressure wave exposure has myriad effects. In our model, the pressure is sufficient to induce mild traumatic brain injury (TBI), primary blast injuries to hollow organs such as the lungs, and increase the complexity of associated extremity injuries through endothelial activation [48–52]. Our investigation of the encapsulated levels of proinflammatory mediators following blast injury reveals an important observation regarding the acute inflammatory status following exposure to a blast overpressure wave. To our knowledge, this study is the first to identify that while conventional immunoassay measurements reveal non-significant early changes in levels of proinflammatory mediators, there is a profound undetected response occurring in exosomes. A study of pre- and post-deployment serum cytokine measurements in military members meeting qualifications for blast-related TBI showed no significant differences in a majority of inflammatory mediators, despite meeting the clinical requirements for TBI [53]. As demonstrated clinically, and in previous studies using our model, the addition of blast increases delayed inflammatory-related healing complications such as heterotopic ossification, suggesting an important role of blast in activation of inflammatory signaling [37, 54]. Furthermore, we show that the addition of blast prior to polytraumatic injuries results in heightened inflammatory responses leading to increased end organ damage. Results from these studies indicate that blast injury induced exosome associated mediators may play a critical role in the early activation of the immune response and could possibly be earlier drivers of down-stream neuroinflammatory events.

Our polytraumatic models recapitulate complex combat injuries with documentation of increased complications related to post-traumatic inflammation [55–57]. Management of the inflammatory response is critical for appropriate resolution, prevention of secondary complications, and transition to a pro-healing phenotype [11, 34]. In relation to the mediators we assessed, previous clinical characterizations of post-traumatic injury inflammatory status using conventional immunoassays rely heavily on IL-6, IL-10, and TNF-α [23]. In polytrauma these mediators are predictive of the development of MODS and MOF. IFN-γ, IL-1β, and IL-4 are reported to have inconsistent value, or minimal data available to assess value in predicting outcome [23]. IL-6 elevations precede other markers of injury severity such as the hepatic acute phase protein c-reactive protein (CRP) [20]. IL-6 levels are noted to have an early increase, followed by a steady decrease in the first 24 h following trauma [20]. Specifically in rats, IL-6 and CINC-1 (CXCL1) are reported as appropriate surrogate inflammatory markers in acute inflammation, peaking in the first 24 h, consistent with our data, where magnitude correlates with severity of injury [58]. An important observation made in assessment of cytokines for predicting patient outcome is that the interplay of different cytokines (IL-6 and IL-10) and ratios of specific cell-associated signaling molecules (Th1-associated vs Th2-associated) are important factors in stratification of patient risk for development of secondary inflammation-induced complications [21]. This observation is particularly relevant to our data which shows increased total levels of IL-10 and TNF-α when accounting for encapsulated cytokines.

The improved prognostic value for added consideration of EC mediators in our model appears to be about 3 h using IL-6 as a surrogate marker of inflammatory status. By 6 h, the majority of IL-6 is found in the FC form and captured using conventional methods. The molecular re-distribution phenomenon precludes the 1 h data being improved by EC IL-6 inclusion, possibly because the cytokine has been delivered to target cells for consumption. Based on the clinical chemistry results, B + COI + IRI + dHLA was deemed the most severe injury pattern, but using conventional parameters, the level of IL-6 is highest in the serum of the B + COI + dHLA cohorts, whereas B + COI + IRI + dHLA is similar to COI + IRI. When the EC IL-6 is taken into account, there is an earlier significant increase in the B + COI + IRI + dHLA that is sustained for a longer period of time. Whereas the COI + IRI and B + COI + dHLA groups show decreases in levels of EC IL-6, the B + COI + IRI + dHLA groups show increasing EC IL-6 compared to baseline in addition to increased FC IL-6, suggesting a potential role for EC IL-6 in sustained inflammatory response and systemic injury in severe polytrauma. This prognostic window may be improved to 1 h post-injury when using EC IL-1β as a prognostic indicator. IL-1β levels are much higher in the calculated total cytokine measures compared to standard at all timepoints, due to a consistently large population of EC IL-1β. Our data may offer returned value to measuring IFN-γ and IL-4 in post-traumatic injury inflammatory status, if decreased encapsulation levels can be consistently quantified across patient populations. The more severe injury patterns demonstrate a consistent, and sustained decrease in the proportion of encapsulated mediator. Expanding the number of reliable mediators for assessing patient status would also give a more complete picture of the immune and inflammatory response.

The post-injury molecular re-distribution from EC to FC/MA in the polytrauma models suggests that there is a stimulus dependent change in exosome transport of proinflammatory mediators following traumatic injury. Given the differences in this change across injury pattern, this effect appears to go beyond the binary “injured” vs “not-injured” and linked to specific trauma patterns. Increasing encapsulated mediators may indicate patient predisposition to a larger inflammatory response in subsequent insults, based on the molecular re-distribution phenomenon found in the polytrauma models including blast. Further, B + COI + IRI + dHLA is the most severe injury pattern, and interestingly shows a rebound increase in encapsulated mediators compared to COI + IRI + HLA and B + COI + dHLA, suggesting an undefined role for exosome encapsulation and transport of circulating mediators in severe, extended inflammatory responses. Given exosomes reflect the metabolic status of the host parental cells, these findings shed light on the role of exosomes and their cargo on immunoregulatory signaling and intercellular communication between cells at different levels and types of traumatic injury and immune activation [34]. The identification of biomarkers in polytrauma patients is important to elucidate mechanisms of pathophysiology, monitor disease progression, and for development of therapeutic targets for timely and precise intervention.

There were some study limitations. The exosome isolation process is not 100% efficient and calculations were necessary to account for dilutional effects, but likely do not fully compensate for the differences between true and observed values. The total mediator levels reported rely on normalized calculated values. Based on these considerations we believe that the true levels of exosome associated mediators are underestimated, and that the actual impact may be greater than what we are reporting. The freely circulating levels may be overestimated in some cases due to the potential for lysis of exosomes to occur as part of the freeze/thaw cycle of serum. At this time, we are unable to make definitive statements regarding whether observed changes in the levels of exosome encapsulated mediators are due to changes in cellular production and packaging or an overall change in cellular release of exosomes. Regardless of these limitations, our results indicate that standard immunoassay measurements are not capturing the complete picture of the post-traumatic inflammatory status. Another important consideration is that we are reporting circulating serum mediator levels, and these do not directly reflect the local wound environment, which may be more appropriately characterized using wound effluent and/or tissue biopsies. Further study limitations were a result of IACUC considerations. We used the minimal number of animals necessary to achieve statistical power analyses and relied on data pooled from cohorts rather than serial longitudinal sampling in individual animals. Because of these constraints, we were also limited in how many sampling timepoints we could accommodate.

## Conclusion

Exosomes as couriers of proinflammatory mediators appear to play a role in orchestrating the progression and outcomes of trauma. Selective encapsulation of mediators and temporal changes in serum compartmentalization suggests a dynamic balance in delivery of inflammatory signaling molecules for modulation of response, and roles for exosomes as effectors of the immune response to trauma. Further studies are warranted to investigate additional timepoints post-injury, and to elucidate the mechanics of mediator packaging and exosome release following traumatic injury.

## Data Availability

The datasets used and/or analyzed during the current study are available from the corresponding author on reasonable request.
